# Implant Maintenance: A Clinical Update

**DOI:** 10.1155/2014/908534

**Published:** 2014-07-09

**Authors:** Minkle Gulati, Vivek Govila, Vishal Anand, Bhargavi Anand

**Affiliations:** ^1^Department of Periodontics, Surendra Dental College & Research Institute, Sri Ganganagar, Rajasthan 335 001, India; ^2^Department of Periodontics, Babu Banarasi Das College of Dental Sciences, Babu Banarasi Das University, Lucknow, Uttar Pradesh 227015, India; ^3^Department of Periodontics, Sarjug Dental College & Hospital, Darbhanga, Bihar 846003, India; ^4^Department of Prosthodontics, Rama Dental College and Hospital and Research Centre, Kanpur, Uttar Pradesh 208025, India

## Abstract

*Introduction*. The differences in the supporting structure of the implant make them more susceptible to inflammation and bone loss when plaque accumulates as compared to the teeth. Therefore, a comprehensive maintenance protocol should be followed to ensure the longevity of the implant. *Material and Method*. A research to provide scientific evidence supporting the feasibility of various implant care methods was carried out using various online resources to retrieve relevant studies published since 1985. *Results*. The electronic search yielded 708 titles, out of which a total of 42 articles were considered appropriate and finally included for the preparation of this review article. *Discussion*. A typical maintenance visit for patients with dental implants should last 1 hour and should be scheduled every 3 months to evaluate any changes in their oral and general history. It is essential to have a proper instrument selection to prevent damage to the implant surface and trauma to the peri-implant tissues. *Conclusion*. As the number of patients opting for dental implants is increasing, it becomes increasingly essential to know the differences between natural teeth and implant care and accept the challenges of maintaining these restorations.

## 1. Introduction

Placement of implant requires an interdisciplinary approach wherein a team of dental implant specialists including oral surgeon, prosthodontist, periodontist, and oral radiologist participate in the planning, execution, and maintenance of the implants to ensure the best possible outcome. Once the implants have been placed in the edentulous region routine maintenance, recall evaluations and radiographs are necessary to insure the long life of these restorations, and this necessitates the team of dental implant specialists to be well versed with the implant maintenance procedures as well, as an implant failure would result in a debate, which would give the profession no credit. These procedures are usually performed at selected intervals to assist the patient in maintaining oral implant health [1]. With time, the emphasis for long-term success of implant has changed from a focus on the surgical phase of treatment to obtaining osseointegration and, now, recently, towards the long-term maintenance health of the peri-implant hard and soft tissues [2, 3]. As the number of patients opting for dental implants as a treatment modality to replace missing teeth continues to grow, it becomes increasingly essential for the dental team to accept the challenges of maintaining these sometimes complex restorations [3]. The long-term success of implants is fundamentally dependent upon both the patient's maintenance of effective home care and on the dental team's administration of professional prophylaxis procedures in the dental office. Hence, patients are considered cotherapists in the maintenance therapy and their contribution is indispensable, especially for the long-term success of dental implants [4]. Minimizing the incidence of implant loss by regular monitoring of the patient and preventing the recurrence of disease progression in previously treated peri-implantitis, patients along with increasing the probability of locating and treating peri-implant pathology in a timely manner are the main therapeutic goals of maintenance therapy [1].

## 2. Material and Method

A comprehensive search to provide scientific evidence supporting the feasibility of various implant care methods was carried out using various online resources like PUBMED, Wiley-Blackwell, Elsevier, Hindawi, and so forth to retrieve relevant studies published since 1985 using the following keywords: “implant maintenance,” “peri-implant,” “implant care,” and “supportive therapy” (and their synonyms). The electronic search by combined mesh term was further augmented by hand search through the following journals and books: Clinical Implant Dentistry and Related Research, Clinical Oral Implants Research, Implant Dentistry, International Journal of Oral & Maxillofacial Implants, International Journal of Oral and Maxillofacial Surgery, International Journal of Periodontics & Restorative Dentistry, International Journal of Prosthodontics, Journal of Clinical Periodontology, Journal of Dental Research, Journal of Periodontology, Journal of Prosthetic Dentistry, Dental Clinics of North America, Misch CE: Contemporary implant dentistry, and Babbush CA (ed.): Dental Implants: The Art and Science. The search was completed by checking references of the relevant review articles and eligible studies for additional useful publications.

## 3. Results

**Figure 1:**
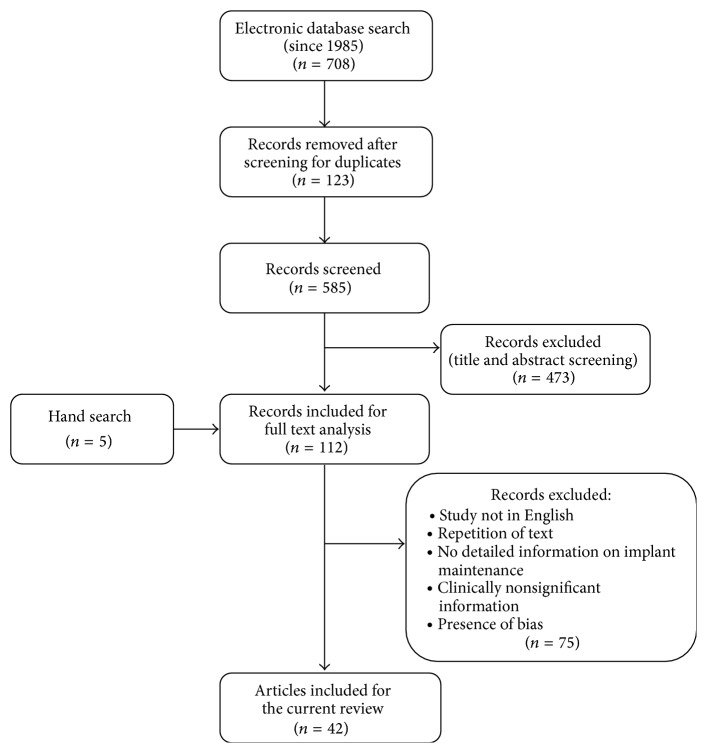
Algorithm of the study selection procedure.

The electronic search yielded 708 titles, whose titles and abstracts were then screened on the basis of their pertinence to the topic “implant maintenance.” Full-text documents were obtained for 112 articles, which were found relevant after going through their abstracts. In addition, 5 publications were also included by hand search, which included 3 chapters. Based upon reading these 117 full-text articles, a total of 42 articles were considered apposite for the discussion and finally included for the preparation of this review article which included 19 clinical research studies (including observational, randomized controlled trial and pilot studies), 19 reviews, 1 case report, and 3 chapters.  Figure 1 outlines the algorithm of the study selection procedure and its results.

The relevant and clinically applicable information gathered from the articles reviewed has been discussed and stated below to update the knowledge of dental clinicians regarding implant maintenance, which is a crucial yet relatively neglected part of implant dentistry.

## 4. Discussion

Long-term success of implant predominantly depends upon the long-term maintenance of the health of the peri-implant hard and soft tissues [2]. A typical maintenance visit for patients with dental implants should include updating patient's medical and dental history, reviewing the patient's oral hygiene and modifying, if necessary, clinical and radiographic examination of the implants and peri-implant tissues, evaluating implant stability, and removing any implant-retained plaque and calculus and setting maintenance intervals [5, 6]. This maintenance visit should last 1 hour and should be scheduled every 3 months.

## 5. Peri-Implant Diagnostic Parameters

The clinical and radiographic parameters routinely used to monitor oral implants during maintenance care should be of high sensitivity and specificity, should be easy to measure, and should yield reproducible data [7]. Though the primary function of a dental implant is to act similar to a natural tooth root and crown, they are fundamentally different from the natural teeth [8]. Therefore, dental indices are often modified for the purpose of dental implant evaluation. The following are various diagnostic parameters to assess peri-implant health.

### 5.1. Plaque and Mucosal Assessment

Accordingly, Mombelli et al. [9] and Apse et al. [10] proposed modified indices for the evaluation of the peri-implant marginal mucosal conditions and plaque assessment (Tables 1 and 2).

### 5.2. Bleeding on Probing (BOP)

Lang et al. in 1994 demonstrated that healthy peri-implant sites were characterized by the absence of bleeding (0%), whereas both peri-implant mucositis and peri-implantitis sites showed substantially increased BOP (67% and 91%, resp.) [11]. Later, Luterbacher and coworkers reported that BOP alone yields higher diagnostic accuracy at implant sites compared with tooth sites [12].

### 5.3. Peri-Implant Probing Depth

The junctional epithelial attachment zone has less attachment strength to the implant and the connective tissue zone has only two fiber groups and neither of those is inserted into the implant. As a result, the probe goes beyond the peri-implant sulcus and reaches closer to the bone; hence, less probing force (0.2-0.3 N) is recommended around implants [13]. Successful implants generally have a probing depth of 3 mm, whereas pocket of 5 mm or more serves as a protected niche for the bacteria and can exhibit signs of peri-implantitis [14]. Bacterial infiltration of the peri-implant sulcus can be avoided by dipping the probe in chlorhexidine prior to its usage [15]. Probing is an appropriate method to assess potential deleterious changes in the peri-implant environment and should be performed every 3 to 4 months for 1 year after prosthesis delivery [8]. However, to avoid interruption during healing and establishment of the soft tissue seal, it should be avoided during the first 3 months after abutment connection [16].

### 5.4. Width of Peri-Implant Keratinized Mucosa

The influence of absence or presence of a zone of keratinized gingiva around teeth and oral implants is still a controversial issue [8]. The presence of keratinized tissue next to an oral implant presents greater benefits than with natural teeth since the keratinized gingiva has more hemidesmosomes and hence provides greater strength to the implant soft tissue interface; also, the submerged implant is less likely to become exposed during the healing process. Moreover, with mobile nonkeratinized tissues, the formation of interdental/implant papillae is completely unpredictable [8].

### 5.5. Peri-Implant Sulcus Fluid Analysis (PISF)

Several biochemical mediators in the PISF have been identified as potential host markers for peri-implant disease activity and progression and their analysis offers a noninvasive means of evaluating the role of host response in peri-implant disease. Niimi and Ueda demonstrated a positive correlation between PISF volume and plaque accumulation as well as degree of peri-implant soft tissue inflammation [17]. Behneke and associates were further able to show an association between PISF volume and the amount of bone resorption [18].

### 5.6. Suppuration

Peri-implantitis is often associated with bleeding, suppuration, increased probing depth, mobility, and bone loss; therefore, suppuration is a definite indicator of the disease activity and indicates the need for anti-infective therapy [19].

### 5.7. Occlusal Evaluation

The occlusal status of the implant and its prosthesis must be evaluated on a routine basis. Any signs of occlusal disharmonies, such as premature contacts or interferences, should be identified and corrected to prevent occlusal overload which can in turn cause a host of problems, including loosening of abutment screws, implant failure, and prosthetic failure [13].

### 5.8. Radiographic Evaluation

A mean crestal bone loss ≥1.5 mm during the first year after loading and ≥0.2 mm/year thereafter has been proposed as one of the major success criteria [20, 21]. Hence, long-term preservation of peri-implant crestal bone height is extremely crucial. In general, the long-cone paralleling technique, supported by positioning devices, is used. Preventive maintenance appointments should be scheduled every 3 to 4 months and a periapical/vertical bitewing radiograph at 6 to 8 months should be compared with the baseline to assess crestal bone changes, which occur often during the first year of loading. These two previous radiographs should be compared with another vertical bitewing radiograph at 1 year. If no changes or unfavorable clinical signs are apparent, subsequent radiographic examinations may be scheduled every 3 years. However, if crestal changes are evident, radiographs must be taken and reviewed every 6 to 8 months until the bone is stable for two consecutive periods, besides stress reduction and hygiene modification [8].

### 5.9. Evaluation of Implant Stability/Mobility

Unlike a tooth, for which mobility is not a primary factor for longevity, mobility is a primary determining factor for implant health. Rigid fixation is usually the first clinical criterion evaluated for a dental implant [8]. The techniques to assess rigid fixation are similar to those used for natural tooth mobility. Two rigid instruments apply a labiolingual force of approximately 500 g. The amplitude of tooth mobility may be rated from 0 to 4 on an implant mobility scale given by Misch (Table 3) [8]. Though the recording of implant mobility may be specific—but it is not a sensitive—clinical parameter in detecting loss of osseointegration, this parameter more likely detects the final stage of osseodisintegration and, therefore, represents a late implant loss [7]. An electronic device (Periotest) has been recommended to monitor initial degrees of implant mobility, but the prognostic accuracy of Periotest value for the diagnosis of peri-implantitis and early signs of implant failure has been criticized because of the lack of resolution, poor sensitivity, and susceptibility to operator variables [22]. Recently, a noninvasive device based on the principles of resonance frequency analysis (RFA) has been developed to measure primary implant stability and to monitor implant stability over time. This method not only evaluates the stiffness of the bone-implant interface but also allows the detection of any increase or decrease in implant stability that otherwise could not be clinically perceived [23–25].

## 6. At-Home Implant Care

Patients with dental implants generally have a history of less-than-ideal home care, resulting in the partially or edentulous state [13]. These patients may moreover have improper oral hygiene practice due to postsurgical fear of causing damage, on the one hand, or overzealous home care trying to stay absolutely plaque free, on the other hand. Either of these situations can lead to detrimental consequences [26]. Therefore, oral hygiene instructions should include detailed verbal guidance and visual demonstration for the long-term success of the implant and its restoration. Also, the oral hygiene techniques and aids used by patients should be reevaluated during every hygiene visit [27]. As demonstrated by Quirynen et al. [28], smooth implant surfaces form less plaque than roughened surfaces. Therefore, it is important to use and recommend those home care aids that will not alter the implant abutment surface and are also safe and effective with daily use [29] (Table 4). The patient must initiate the implant care regimen immediately after surgical placement with one-stage system, after exposure of the implant site in the two-stage system and upon premature exposure of the implant healing screw in the two-stage system [30]. However, during healing periods, when mechanical plaque control is contraindicated, chemical agents (e.g., chlorhexidine) should be used [13].

### 6.1. Brushing

Twice daily cleaning of implants to remove bacterial plaque accumulations should be accomplished using a soft toothbrush such as Nimbus Microfine (Nimbus Dental, Los Altos, CA, USA) or a very gentle power brush [27]. Also motorized toothbrush like Rota-Dent (Pro-Dentec, Professional Dental Technologies, Inc., Batesville, AR), with its patented microfilaments, is very gentle to the tissues, as well as nonabrasive to the abutment, and may be used along with a tapered brush to access the undersurface of connector bars or to aid with interdental cleansing [27, 30]. Patients should be instructed in circular brushing according to the BASS technique using small, soft-bristled brushes [4].

Several automated/sonic tooth brushes with multiple brush tips have also been developed but may result in gingival abrasion from prolonged use. An automated toothbrush, Sonicare, developed by the University of Washington and Optiva Corporation, Bellevue, WA, however, has been shown to not cause hard or soft tissue damage and to effectively reduce the plaque and inflammation around the adjacent periodontal tissues [4]. These brushes are considered superior to a manual toothbrush in removing plaque and they contribute to the improved interproximal cleaning due to the combination of their bristle shape (scalloped) and fluid penetration [4]. A patient with limited dexterity should use a power or sonic toothbrush [31].

In difficult-to-access areas smaller-diameter toothbrush heads such as end-tufted brushes or tapered rotary brushes may be of benefit [13]. An end-tufted brush can be manipulated under hot water to accommodate the shape of the prosthesis and is especially useful in posterior regions where a conventional toothbrush might not reach [30, 31].

### 6.2. Interproximal/Circumferential Cleaning

There are many flosses, interproximal cleaners, and water irrigation systems commercially available and safe for use around implants.

#### 6.2.1. Floss

Floss choice should be based on the clinical indication [27]. The following types of floss may be used to remove interproximal plaque [13, 27, 30, 31].

- Plastic floss, such as ProxiFloss (AIT Dental Inc., Tess Corporation, Eau Claire, WI), is an elastomeric material that bends and flexes to remove plaque or to apply chemotherapeutic agents while preventing the floss from collapsing, snagging, or shredding.
- Braided flossing cord, such as PostCare (John O. Butler Co., USA), is more rigid than conventional floss and suitable for open areas and places where a floss threader may be too fragile to remove denser plaque, debris, and calculus.
- Satin Floss (Oral-B, Procter & Gamble Company, Toronto) or Glide (W. L. Gore & Associates, Inc., Newark, DE) is particularly appropriate for a single tooth implant with intimate tissue adaptation.
- Woven, such as Thornton Bridge & Implant Cleaners (Thornton International, Inc., Norwalk, CT) or GUM Expanding Floss (Sunstar Butler, Foster Avenue, Chicago, IL, USA), is indicated where there are large interproximal spaces or long expanses of a bar retained prosthesis.
- Yarns can be used to access and cleanse larger embrasure spaces and under connector bars, but these should not be considered if there is the possibility of the fibers being retained on rough surfaces or around the restorations.
- Dental Tapes are available in different “widths” and are used to clean the exposed abutment.

Other types include tufted, coated, and gauze thicker dental floss. All of these can be used in a “shoe-shine rag” fashion to facilitate optimal home care procedures around the abutment post [30]. Threader floss may also be needed to access bridgework or around connector bars.

Traditional flossing of the mesial and distal surfaces is required, but it is often indicated to use the floss on the facial/lingual surfaces as well following the looping technique [27]. Dental floss can also be used to deliver antiseptic agents to the implant on a daily basis. With all types of flossing materials, it is important to instruct the patient to gently place floss subgingivally until resistance is met [4].

#### 6.2.2. Interproximal Cleaners

Interdental aids can be selected and recommended considering the size and shape of the embrasure, when patients are unable to use floss. Foam tips, interproximal brushes, and disposable wooden picks are among the many auxiliary devices that can assist in plaque removal and delivering antiseptic rinses to enhance their effectiveness [31].

Interproximal brushes should be chosen based on the interproximal area. Whereas larger spaces can be properly cleaned with a proxy brush such as StaiNo Interdental Brushes (StainNo, LLC, Long Eddy, NY, USA), smaller interdental brushes, such as the sulcabrush or Go-Betweens Cleaners (Sunstar Butler, Foster Avenue, Chicago, IL, USA), are helpful in narrower interproximal spaces [27]. However, caution must be used with interproximal brushes that have an exposed tip of metal wire or if enough pressure is exerted, as that can easily scratch the abutment's titanium surface. Hence, in case of implant, an interproximal brush with a plastic-coated wire is usually recommended [4]. Chemotherapeutic agents can also be applied interdentally and site specifically using foam tips and Proxi-Tip (AIT Dental Inc., Tess Corporation, Eau Claire, WI) which acts as an interproximal brush and rubber tip in one design [30].

#### 6.2.3. Water Irrigation

A water irrigation unit such as the Hydro Floss (Hydro Floss, Inc.) is also beneficial in implant maintenance. However, care must be taken to direct the stream interproximally and horizontally between implants, as improper positioning can cause inadvertent damage to the peri-implant seal and bacteremia [27, 30].

### 6.3. Locally Applied Chemotherapeutics

For implant patients especially those prone to occasional tissue inflammation, an at-home regimen of daily cleansing with chemotherapeutic agents in the form of rinses, gels, or solutions is extremely essential. Site-specific application of chlorhexidine or another anti-infective solution is better than rinsing, as it will not only specifically treat the target area but also help to remove plaque, control staining, and decrease tartar buildup at the same time [32]. It has been documented that topical antimicrobials such as products containing chlorhexidine digluconate (0.12%), plant alkaloids, or phenolic agents produce minimal implant surface alterations [29].

### 6.4. Intraoral Camera

The intraoral camera can be used for periodic tissue checks by the patient or to check the effectiveness of their oral care routine and can be connected to a patient's television. Patient can pinpoint any food lodgment, redness, swelling, or other signs around the implant and severe infections can be avoided by taking early preventive steps [27].

## 7. Professional Hygiene Care

Implants necessitate intensive care that goes far beyond mere brushing of teeth. Natural teeth are anchored to the socket via periodontal ligament, which has an inherent protective defense mechanism, and hence are better protected against outside attacks than the implant. Despite the long-term predictability of implants, complications do occur in a percentage of cases and can ultimately result in loss of implants and failure of prostheses. Adequate maintenance programs and regular professional hygiene care for patients with dental implants as well as treating peri-implant pathology in a timely manner can minimize and prevent implant loss/failure due to such complications [1] (Table 4).

### 7.1. Instrument Selection

The instruments selected should not be bulky and should be disposable or able to be sterilized, effective in removing plaque and calculus without damaging the implant surface, cost effective, easy to use, and adaptable in the implant sulcus [30].

#### 7.1.1. Scaling

Removal of calculus and plaque, if present, is indicated for implants at a hygiene visit. Metallic instruments, such as stainless steel, should not be used to probe or scale dental implants as they can scratch, roughen, contaminate, or cause a galvanic reaction at the implant-abutment interface that will further make the titanium surface more susceptible to bacterial plaque and calculus buildup, increasing the possibility of peri-implant inflammation [3, 27, 33].

Plastic instruments produce insignificant alteration of the implant surface and are, thus, recommended for scaling implants, even though residues from the instruments are left behind [13, 34]. Plastic instruments reinforced with graphite and gold-plated curettes are more rigid and can be sharpened and can as well be used [13]. However, caution must be exercised when sharpening these gold-plated instruments and when using them on rough surface, as the gold surface could be chipped and worn down, respectively, exposing the underlying alloy and leaving an unsuitable surface [3, 13]. Upon insertion of the instrument, the blade should be closed against the abutment and then opened past the deposit, engaging it apically with the stroke extending coronally. Depending on the location of the deposit, horizontal, oblique, or vertical, short working strokes and light pressure should be used to prevent trauma to the delicate peri-implant sulcus. Prostheses can sometimes limit access of the scaler, and, in such cases, an ultrasonic or sonic scaler covered with a plastic sleeve can be used to remove deposits [31]. The nonporous titanium surface calculus that forms around implants tends to be softer than calculus adhering to a natural tooth and is mostly supragingival. Occasionally, harder deposit around an implant may be found, which can be removed using a product like SofScale (Dentsply Professional, York, PA, USA) before scaling to further reduce the risk of scratching the implant during calculus removal [27].

Examples of some of the commercially available scalers and curettes for cleaning implant surfaces are Implacare (Hu-Friedy, IL, USA) made of Plasteel, a high grade resin; 3i-Implant Innovations, Inc., (West Palm Beach, FL) implant scaler made up of high-tech plastic; Steri-Oss scaler system (Yorba Linda, California) constructed of graphite-reinforced nylon; Implant Cleaning Kits (Brevet Inc., Irvine, CA); and so forth [30].

### 7.2. Polishing

The main indication for polishing an implant is for plaque removal, since titanium surface of an implant abutment is highly polished and with proper care will rarely loose its manufacturer's polished finish [27]. 3i-Implant Innovations, Inc. (West Palm Beach, FL) offers an implant polishing kit containing Abutment Glo polishing paste and a variety of polishing cups and soft-tipped brushes [4, 30].

The prosthesis and abutments may be selectively polished with a rubber cup and nonabrasive polishing paste such as aluminum oxide, tin oxide, APF-free prophy paste, and low-abrasive dentifrice after hard deposits have been removed [4, 27, 35]. An antibacterial solution such as chlorhexidine may be used, when no polishing agent is desired [27]. When only soft debris is present, deplaquing the surface is beneficial. Coarse abrasive polishing pastes, flour or pumice for polishing, are contraindicated, as is air polishing [36, 37].

Air polishing of implant components remains controversial. Whereas some researchers suggest that the air-abrasive units like PROPHYPearls (KaVo Dental, Charlotte, North Carolina Area, USA) and Jet Fresh (Hongchang Dental Equipment Co., Ltd., Hubei, China) are safe and effective in removing deposits [38–41], others contraindicate the use of air polishing as it can cause damage to the porcelain or composite materials [27], can create random pitting or undulating wave-type of surface irregularities on the titanium [29], and may detach the soft tissue connection from the implant due to air pressure leading to emphysema [3].

### 7.3. Locally Applied Chemotherapeutics

Early intervention with a locally applied antibiotic or antimicrobial, such as Arestin Atridox, PerioChip, or Dentomycin or subgingival irrigation with an antiseptic agent such as peroxide, Listerine, or chlorhexidine using a plastic irrigation tip may help to slow or reverse the inflammation [32, 33]. A cannula should have nonmetallic, rounded tip with side escape portals, and care should be taken while inserting it to the base of the implant sulcus to prevent fluid distention into surrounding tissues and to avoid gouging the surface [30, 33]. It is also wise to use a neutral sodium fluoride in a patient with dental implants because certain acidic fluorides can alter titanium [33]. A study by Renvert et al. on nonsurgical mechanical treatment on sites with peri-implantitis lesions with microencapsulated minocycline (Arestin) and 0.12% chlorhexidine gel found reductions of pocket depths and bleeding on probing for as long as 12 months [42].

## 8. Summary

Dental implants require constant maintenance and monitoring, which further involves assessment of the patient's general and oral health, professional implant maintenance, and diligent patient home care as critical factors that will ensure the long-term success of implants and a predictable replacement for natural teeth.

## Figures and Tables

**Table 1:** Peri-implant plaque assessment index.

Score	Mombelli et al. [9] (mPI)
0	No detection of plaque
1	Plaque only recognized by running a probe across the smooth marginal surface of the implant
2	Plaque which can be seen by the naked eye
3	Abundance of soft matter

Reference [9].

**Table 2:** Peri-implant marginal mucosal indices.

Score	Apse et al. [10]	Mombelli et al. [9] (mGI)
0	Normal mucosa	No bleeding when a periodontal probe is passed along the mucosal margin adjacent to the implant
1	Minimal inflammation along with color change and minor edema	Isolated bleeding spots visible
2	Moderate inflammation with redness, edema, and glazing	Blood which forms a confluent red line on mucosal margin
3	Severe inflammation with redness, edema, ulceration, and spontaneous bleeding without probing	Heavy or profuse bleeding

References [9, 10].

**Table 3:** Clinical implant mobility scale.

Scale	Description
0	Absence of clinical mobility with 500 g in any direction
1	Slight detectable horizontal movement
2	Moderate visible horizontal mobility up to 0.5 mm
3	Severe horizontal movement greater than 0.5 mm
4	Visible moderate to severe horizontal and any visible vertical movement

Reference [10].

**Table 4:** Implant maintenance summarized.

At-home implant care	Professional hygiene care
*Brushing* Soft manual toothbrush Motorized tooth brush/power brush Automated/sonic tooth brush End-tufted brush Tapered rotary brush	*Scaling andcurettage* Plastic instruments Plastic instruments reinforced with graphite Gold-plated curettes Ultrasonic or sonic scaler covered with a plastic sleeve

*Interproximal/circumferential cleaning*: (i) Floss Plastic floss Braided flossing cord Satin floss Woven floss Yarns dental tapes (ii) Interproximal cleaners Foam tips Interproximal brushes with a plastic coated wire Disposable wooden picks	*Polishing* (i) Rubber cup with a nonabrasive polishing paste Such as aluminum oxide, tin oxide, APF-free prophy paste, and low-abrasive dentifrice (ii) Air polishing (Use remains controversial)

*Locally applied chemotherapeutics* For example: chlorhexidine digluconate (0.12%), plant alkaloids, or phenolic agents	*Locally applied chemotherapeutics* Such as Arestin, Atridox, PerioChip, or Dentomycin

*Water irrigation* For example: Hydro Floss	*Subgingival irrigation* Antiseptic agents such as Peroxide, Listerine, or Chlorhexidine using a plastic irrigation tip

## References

[1] Cohen R. E. (2003). Position paper: periodontal maintenance. *Journal of Periodontology*.

[2] Silverstein L. H., Kurtzman G. M. (2006). Oral hygiene and maintenance of dental implants. *Dentistry Today*.

[3] Kurtzman G. M., Silverstein L. H. (2007). Dental implants: oral hygiene and maintenance: implant. *Dentistry Today*.

[4] Garg A. K., Duarte F., Funari K. (1997). Hygeinic maintenance of dental implants. *Journal of Practical Hygiene*.

[5] Wilson T. G. (1996). A typical maintenance visit for patients with dental implants. *Periodontology 2000*.

[6] Wilson T. G. (1990). Maintaining periodontal treatment. *The Journal of the American Dental Association*.

[7] Salvi G. E., Lang N. P. (2004). Diagnostic parameters for monitoring peri-implant conditions. *International Journal of Oral and Maxillofacial Implants*.

[8] Misch C. E., Misch C. E. (2007). An implant is not a tooth: a comparison of periodontal indices. *Contemporary Implant Dentistry*.

[9] Mombelli A., van Oosten M. A. C., Schürch E., Land N. P. (1987). The microbiota associated with successful or failing osseointegrated titanium implants. *Oral Microbiology and Immunology*.

[10] Apse P., Zarb G. A., Schmitt A., Lewis D. W. (1991). The longitudinal effectiveness of osseointegrated dental implants. The Toronto study: peri-implant mucosal response. *The International Journal of Periodontics & Restorative Dentistry*.

[11] Lang N. P., Wetzel A. C., Stich H., Caffesse R. G. (1994). Histologic probe penetration in healthy and inflamed peri-implant tissues. *Clinical Oral Implants Research*.

[12] Luterbacher S., Mayfield L., Brägger U., Lang N. P. (2000). Diagnostic characteristics of clinical and microbiological tests for monitoring periodontal and peri-implant mucosal tissue conditions during supportive periodontal therapy (SPT). *Clinical Oral Implants Research*.

[13] Humphrey S. (2006). Implant maintenance. *Dental Clinics of North America*.

[14] Buser D., Weber H. P., Lang N. P. (1990). Tissue integration of non-submerged implants. 1-year results of a prospective study with 100 ITI hollow-cylinder and hollow-screw implants. *Clinical Oral Implants Research*.

[15] Mortilla L. D. T., Misch C. E., Suzuki J. B. (2008). The dental hygienist’s role in implant evaluation & assessment. *Journal of Practical Hygiene*.

[16] Bauman G. R., Mills M., Rapley J. W., Hallmon W. H. (1992). Clinical parameters of evaluation during implant maintenance. *The International Journal of Oral & Maxillofacial Implants*.

[17] Niimi A., Ueda M. (1995). Crevicular fluid in the osseointegrated implant sulcus: a pilot study. *The International Journal of Oral & Maxillofacial Implants*.

[18] Behneke A., Behneke N., D’Hoedt B., Wagner W. (1997). Hard and soft tissue reactions to ITI screw implants: 3-year longitudinal results of a prospective study. *International Journal of Oral and Maxillofacial Implants*.

[19] Klinge B., Hultin M., Berglundh T. (2005). Peri-implantitis. *Dental Clinics of North America*.

[20] Albrektsson T., Zarb G., Worthington P., Eriksson A. R. (1986). The long-term efficacy of currently used dental implants: a review and proposed criteria of success. *The International Journal of Oral & Maxillofacial Implants*.

[21] Smith D. E., Zarb G. A. (1989). Criteria for success of osseointegrated endosseous implants. *The Journal of Prosthetic Dentistry*.

[22] Meredith N. (1998). Assessment of implant stability as a prognostic determinant. *International Journal of Prosthodontics*.

[23] Friberg B., Sennerby L., Linden B., Gröndahl K., Lekholm U. (1999). Stability measurements of one-stage Brånemark implants during healing in mandibles a clinical resonance frequency analysis study. *International Journal of Oral and Maxillofacial Surgery*.

[24] Friberg B., Sennerby L., Meredith N., Lekholm U. (1999). A comparison between cutting torque and resonance frequency measurements of maxillary implants: a 20-month clinical study. *International Journal of Oral and Maxillofacial Surgery*.

[25] Heo S. J., Sennerby L., Odersjö M., Granström G., Tjellström A., Meredith l N. (1998). Stability measurements of craniofacial implants by the means of resonance frequency analysis. A clinical pilot study. *The Journal of Laryngology and Otolog*.

[26] Meffert R. M., Langer B., Fritz M. E. (1992). Dental implants: a review. *Journal of Periodontology*.

[27] Goldstein R. E., Nimmons K. J. (2005). The expanding esthetic practice: implant maintenance—part 2. *Contemporary Esthetics & Restorative Practice*.

[28] Quirynen M., van der Mei H. C., Bollen C. M. (1993). An *in vivo* study of the influence of the surface roughness of implants on the microbiology of supra and subgingival plaque. *Journal of Dental Research*.

[29] Thomson-Neal D., Evans G. H., Meffert R. M. (1989). Effects of various prophylactic treatments on titanium, sapphire, and hydroxyapatite-coated implants: an SEM study. *The International Journal of Periodontics & Restorative Dentistry*.

[30] Mortilla L. D. T., Babbush C. A. (2001). Hygiene and soft tissue management: the hygienist’s perspective. *Dental Implants: The Art and Science*.

[31] Sison G. (2003). Implant maintenance and the dental hygienist. *Access*.

[32] Goldstein R. E., Nimmons K. J. (2005). The expanding esthetic practice: implant maintenance—part 1. *Contemporary Esthetics & Restorative Practice*.

[33] Suzuki J. B., Misch C. E., Bronstein D., Mortilla L. D. T., Misch C. E. (2007). Maintenance of dental implants: implant quality of health scale. *Contemporary Implant Dentistry*.

[34] Walsh L. J. (2007). Implant hygiene: clues, caveats and cautions. *Australasian Dental Practice*.

[35] Matarasso S., Quaremba G., Coraggio F., Vaia E., Cafiero C., Lang N. P. (1996). Maintenance of implants: an *in vitro* study of titanium implant surface modifications subsequent to the application of different prophylaxis procedures. *Clinical Oral Implants Research*.

[36] Bergendal T., Forsgren L., Kvint S., Löwstedt E. (1990). The effect of an airbrasive instrument on soft and hard tissues around osseointegrated implants. A case report. *Swedish Dental Journal*.

[37] Rapley J. W., Swan R. H., Hallmon W. W., Mills M. P. (1990). The surface characteristics produced by various oral hygiene instruments and materials on titanium implant abutments. *The International Journal of Oral & Maxillofacial Implants*.

[38] Augthun M., Tinschert J., Huber A. (1998). *In vitro* studies on the effect of cleaning methods on different implant surfaces. *Journal of Periodontology*.

[39] Mengel R., Buns C.-E., Mengel C., Flores-de-Jacoby L. (1998). An *in vitro* study of the treatment of implant surfaces with different instruments. *International Journal of Oral and Maxillofacial Implants*.

[40] Meschenmoser A., D’Hoedt B., Meyle J. (1996). Effects of various hygiene procedures on the surface characteristics of titanium abutments. *Journal of Periodontology*.

[41] Homiak A. W., Cook P. A., DeBoer J. (1992). Effect of hygiene instrumentation on titanium abutments: a scanning electron microscopy study. *The Journal of Prosthetic Dentistry*.

[42] Renvert S., Lessem J., Dahlén G., Lindahl C., Svensson M. (2006). Topical minocycline microspheres versus topical chlorhexidine gel as an adjunct to mechanical debridement of incipient peri-implant infections: a randomized clinical trial. *Journal of Clinical Periodontology*.

